# Integrated Analysis of Multiple Microarray Studies to Identify Novel Gene Signatures in Non-alcoholic Fatty Liver Disease

**DOI:** 10.3389/fendo.2019.00599

**Published:** 2019-08-30

**Authors:** Xi Jia, Tianyu Zhai

**Affiliations:** ^1^Department of Endocrinology, Jinshan Hospital, Fudan University, Shanghai, China; ^2^Department of Endocrinology and Metabolism, Zhongshan Hospital, Fudan University, Shanghai, China

**Keywords:** non-alcoholic fatty liver disease, microarray, differentially expressed genes, robust rank aggregation, integrated analysis

## Abstract

**Background:** Non-alcoholic fatty liver disease (NAFLD) is a well-known cause of liver dysfunction and has become a common chronic liver disease in many countries. However, the intrinsic molecular mechanisms underlying the pathogenesis of NAFLD have not yet been fully elucidated.

**Methods:** We obtained the gene expression datasets of NAFLD through the Gene Expression Omnibus (GEO) database. Subsequently, robust rank aggregation (RRA) method was used to identify differentially expressed genes (DEGs) between NAFLD patients and controls. Gene functional annotation and PPI network analysis were performed to explore the potential function of the DEGs. Finally, we used a sequencing dataset GSE126848 to validate our results.

**Results:** In this study, GSE48452, GSE66676, GSE72756, GSE63067, GSE89632, and GSE107231 were included, including 125 NAFLD patients and 116 control patients. The RRA integrated analysis determined 96 significant DEGs (50 up-regulated and 46 down-regulated) and the most significant gene aberrantly expressed in NAFLD was ENO3 (*P*-value = 7.17E-05), followed by CYP7A1 (*P*-value = 9.04E-05), and P4HA1 (*P*-value = 1.67E-04). Carboxylic acid metabolic process (GO:0019752; *P*-value = 1.39E-03) was the most significantly enriched for biological process in GO (gene ontology) analysis. KEGG pathway enrichment analysis showed that steroid hormone biosynthesis (hsa00140; *P*-value = 6.68E-03) and PPAR signaling pathway (hsa03320; *P*-value = 9.95E-03) were significantly enriched. Based on the results of the PPI and the results of the RRA, we finally defined the four most critical genes as the hub genes, including ENO3, CYP7A1, P4HA1, and CYP1A1.

**Conclusions:** Our integrated analysis identified novel gene signatures and will contribute to the understanding of comprehensive molecular changes in NAFLD.

## Introduction

Non-alcoholic fatty liver disease (NAFLD) is a well-known cause of liver dysfunction over the world and is closely correlated with obesity, hyperglycemia, and hyperlipidemia (1, 2). NAFLD represents a spectrum of liver disorders that includes simple steatosis and non-alcoholic steatohepatitis (NASH), progressing to cirrhosis, and even hepatocellular carcinoma (HCC) (3). To date, NAFLD has become a common chronic liver disease in many countries, affecting almost 30% of the general population (3, 4), and is currently the third most common indication for liver transplantation in the USA (5). Some studies have attempted to elucidate the effective or causal effects of oxidative stress, adipocytokine production/release, intra-hepatic insulin resistance, fat accumulation, and innate immune system activation in the pathogenesis of NAFLD (3). However, the intrinsic molecular mechanisms underlying the pathogenesis of NAFLD have not yet been fully elucidated, and more research is needed to provide deeper understanding and to explore more advantageous therapeutic targets.

Identifying gene-specific expression patterns has been useful in the understanding of pathogenic mechanisms or therapeutic assessment for multiple diseases, NAFLD included (6, 7). In the past few years, microarray technology has been widely used for gene expression profiling in liver tissue from NAFLD patients or experimental animals. However, there are some inconsistencies in those microarray studies, such as different analysis platforms, data outliers, sample sizes, and sources. The robust rank aggregation (RRA) approach has been used to select differentially expressed mRNA profiles based on multiple datasets in various diseases, such as cancer and autoimmune disease. To the best of our knowledge, previous researches of NAFLD have not used the RRA method to identified differentially expressed genes (DEGs), which facilitated this study.

Thus, we conducted a gene expression meta-analysis between NAFLD liver tissues and control liver tissues using integrated bioinformatics methods. In addition, based on the result of this analysis, hub genes identification in the DEGs and gene enrichment and pathway annotation analysis were also performed.

## Materials and Methods

### Microarray Datasets of NAFLD

We obtained the gene expression datasets of NAFLD through the Gene Expression Omnibus (GEO) database (http://www.ncbi.nlm.nih.gov/) (8). We systematically searched the microarray studies by using the terms: “Fatty liver,” “Non-alcoholic,” “Gene expression,” “Homo sapiens,” and “Microarray.” Datasets were included according to the following eligibility criteria: (1) Containing at least 10 total samples; (2) Containing at least five cases and at least five controls; (3) Raw data or gene expression profiling by array were available in GEO.

### Datasets Analyses

First, we downloaded the gene expression matrix and related annotation document for each array dataset from GEO database, and used corresponding annotation document to map the microarray probes to gene symbols. If multiple probes mapped to the same symbol, the mean value was adopted. The 6 NAFLD expression microarray datasets were all standardized by quantiles. The DEGs were determined between NAFLD tissues and normal control liver tissues in each microarray by using the “limma” (linear models for microarray data) R package. The |log2 fold change (FC)| > 0.5 and *P*-value < 0.05 were regarded as the cut-off criteria to determine DEGs.

### RRA Analysis

To minimize the inconsistencies and to integrate the results from several microarray studies, RRA method was adopted to identify robust DEGs, which is an effective tool to integrate multiple arrays outcomes (9, 10). Before RRA analysis, we obtained up-ranked and down-ranked gene lists of each dataset which were generated by expression fold change between cases and controls. The “Robust Rank Aggregation” R package was used to integrate all the ranked gene lists of datasets. The adjusted *P*-value in the RRA tool indicate the possibility of ranking high of each gene in the final gene list. Genes with *P*-value < 0.05 and the fold change >0.5 were considered as significant genes.

### Functional and Pathway Enrichment Analysis

Database for Annotation, Visualization, and Integrated Discovery (DAVID, http://david.abcc.ncifcrf.gov/) (11–13) is regarded as the most common functional annotation tool, which was used for gene ontology (GO) functional enrichment analysis and Kyoto Encyclopedia of Genes and Genomes (KEGG) pathway analysis. We uploaded the significant genes in RRA analysis to investigate the potential functions. *P*-value < 0.05 and false discovery rate (FDR) <0.05 was regarded as the cut-off criteria.

### Protein-Protein Interaction (PPI) Network Analysis

Hub genes are usually deemed to be functionally critical and highly interconnected with other genes. We uploaded significant genes in RRA analysis to the STRING database (http://www.string-db.org/) and HIPPIE database (http://cbdm.uni-mainz.de/hippie/), then chose confidence >0.4 to perform the PPI network analysis. We synthesized STRING and HIPPIE methods to find the genes with the top 10 connectivity, and matched the results with the top 10 DEGs defined by RRA, and finally obtained the hub gene. The PPI network diagrams of STRING database were plotted by Cytoscape software. In the Cytoscape, each node represents a gene or protein, and the edge between nodes represents the interaction of the molecules.

### RRA and Hub Genes Validation Study

Previous studies have found that arrays also have the risk of cross-hybridization, while RNA-seq data is highly replicable, thus providing a more accurate estimate of gene expression than arrays (14, 15). Therefore, we further conducted RRA validation studies by using data from a RNA-seq dataset (GSE 126848, including 31 NAFLD patients, 14 healthy controls). The RNA-seq reading count data was analyzed using edgeR function to identify DEGs. Genes with *P*-value < 0.05 were considered to be significant.

### Ethical Declaration

All of the data used in this study were obtained from public databases. This study does not contain any studies associated with animals or humans.

## Results

### Information of Included Microarrays

According to the previously established inclusion criteria, GSE48452, GSE66676, GSE72756, GSE63067, GSE89632, and GSE107231 were included in this study. There are 125 NAFLD patients (53 patients with non-alcoholic steatohepatitis and 72 patients with simple steatosis) and 116 controls (non-NAFLD patients, including 89 normal subjects and 27 healthy obese subjects) in these six datasets. The detailed information of these datasets was shown in Table 1.

**Table 1:** Characteristics of the included microarray datasets.

**GSE ID**	**Participants**	**Tissues**	**Analysis type**	**Platform**	**Year**
GSE48452	32 cases and 41 controls	Liver	Array	GPL11532	2013
GSE63067	11 cases and 7 controls	Liver	Array	GPL570	2014
GSE66676	33 cases and 34 controls	Liver	Array	GPL6244	2017
GSE72756	5 cases and 5 controls	Liver	Array	GPL16956	2015
GSE89632	39 cases and 24 controls	Liver	Array	GPL14951	2016
GSE107231	5 cases and 5 controls	Liver	Array	GPL20115	2017

### Identification of DEGs in NAFLD

**Figure 1:**
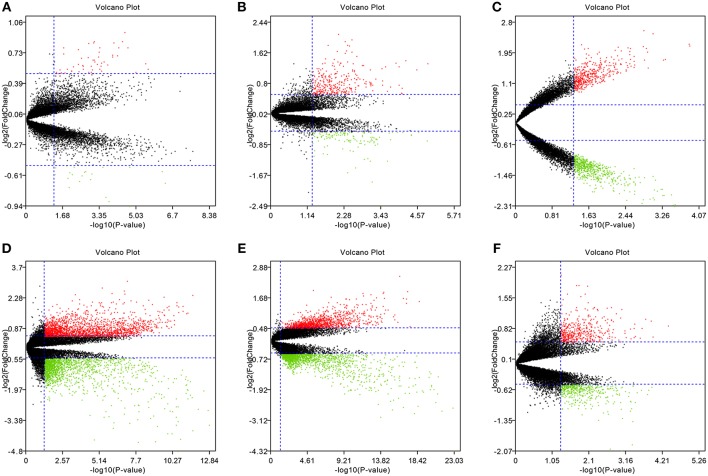
Volcano plots of the six microarrays. Red points represented up-regulated genes, while green points represented down-regulated genes. Black points represented genes with no significant difference. **(A)** GSE48452, **(B)** GSE63067, **(C)** GSE66676, **(D)** GSE72756, **(E)** GSE89632, **(F)** GSE107231.

To eliminate individual differences between samples, all six of these microarray data sets were first standardized by quantiles. The results are shown in Supplementary Figure 1, all samples in each dataset achieved homogeneity which was acceptable. The DEGs were screened out by using the “limma” package in R software according to the cut-off criteria. The volcano plots of the six microarrays were shown in Figure 1.

### Results in the RRA Integrated Analysis

**Figure 2:**
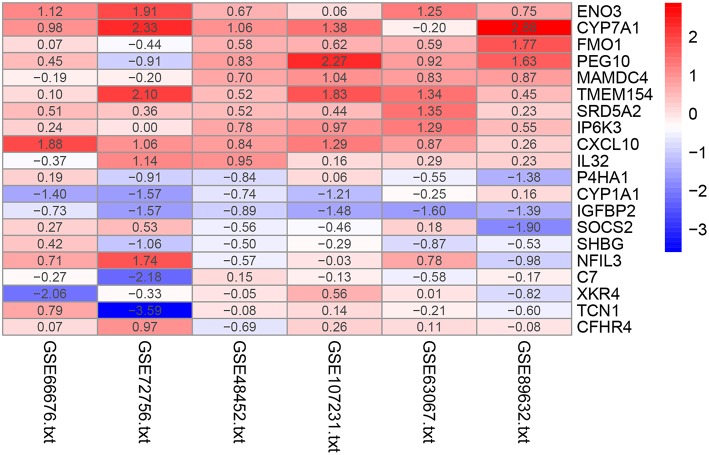
Heatmap of the top 10 up- and down-regulated genes in the RRA analysis. Red represents high expression of genes in patients with NAFLD, while blue represents low expression of genes in patients with NAFLD.

The RRA method assumes that each gene is randomly ordered in each dataset. The smaller the *P*-value in the RRA results, the higher gene ranks and the credibility of gene differential expression. Ninety-six significant DEGs (50 up-regulated and 46 down-regulated) were determined through the integrated analysis (Supplementary Table 1). The heatmap of the top 10 up- and down-regulated genes was shown in Figure 2. The top 10 significant gene aberrantly expressed in NAFLD included five up-regulated genes [ENO3 (*P* = 7.17E-05), CYP7A1 (*P* = 9.04E-05), FMO1 (*P* = 6.57E-04), PEG10 (*P* = 8.95E-04), and MAMDC4 (*P* = 1.87E-03)] and five down-regulated genes [P4HA1 (1.67E-04), CYP1A1 (*P* = 2.51E-04), IGFBP2 (*P* = 3.27E-04), SOCS2 (*P* = 5.1E-04), and SHBG (*P* = 1.50E-03)].

### Functional Annotation

**Figure 3:**
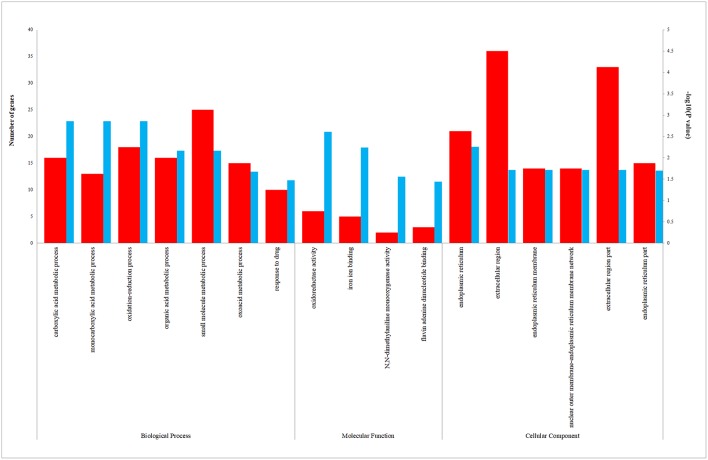
The enriched biological process (BP), molecular function (MF), and cellular component (CC) terms in GO analysis. The red column represents the number of genes enriched in GO term. The blue column represents the –log_10_(*P*-value) of GO term.

We uploaded the 96 DEGs to perform the GO (including biological process, molecular function and cellular component) analysis and KEGG analysis. The outcomes revealed that carboxylic acid metabolic process (GO:0019752; *P*-value = 1.39E-03) was the most significantly enriched for biological process, followed by monocarboxylic acid metabolic process (GO:0032787; *P*-value = 1.39E-03), oxidation-reduction process (GO: 0055114; *P*-value = 1.39E-03) and so on (Table 2). In terms of the molecular function and cellular component, the results were shown in Figure 3. Furthermore, KEGG pathway enrichment analysis showed that steroid hormone biosynthesis (hsa00140; *P*-value = 6.68E-03) and PPAR signaling pathway (hsa03320; *P*-value = 9.95E-03) were significantly enriched, as shown in Table 3 and Supplementary Figure 2.

**Table 2:** GO analysis of DEGs associated with NAFLD in the RRA analysis.

**Term**	**Description**	**Gene count**	***P*-value**
GO.0019752	Carboxylic acid metabolic process	16	1.39E-03
GO.0032787	Monocarboxylic acid metabolic process	13	1.39E-03
GO.0055114	Oxidation-reduction process	18	1.39E-03
GO.0006082	Organic acid metabolic process	16	6.87E-03
GO.0044281	Small molecule metabolic process	25	6.87E-03
GO.0043436	Oxoacid metabolic process	15	2.12E-02
GO.0042493	Response to drug	10	3.34E-02

*GO, gene ontology; DEGs, differentially expressed genes*.

**Table 3:** KEGG analysis of DEGs associated with NAFLD in the RRA analysis.

**Pathway**	**ID**	**Count**	**Genes**	***P*-value**
Steroid hormone biosynthesis	hsa00140	4	CYP1A1, CYP7A1, HSD17B12, SRD5A2	6.68E-03
PPAR signaling pathway	hsa03320	4	ACSL1, PLIN1, CYP7A1, FABP4	9.95E-03
p53 signaling pathway	hsa04115	3	TP53I3, IGF1, GADD45B	7.36E-02
Bile secretion	hsa04976	3	SLCO1A2, CYP7A1, ABCB4	7.74E-02

### PPI Network Analysis and Identification of Hub Gene

**Figure 4:**
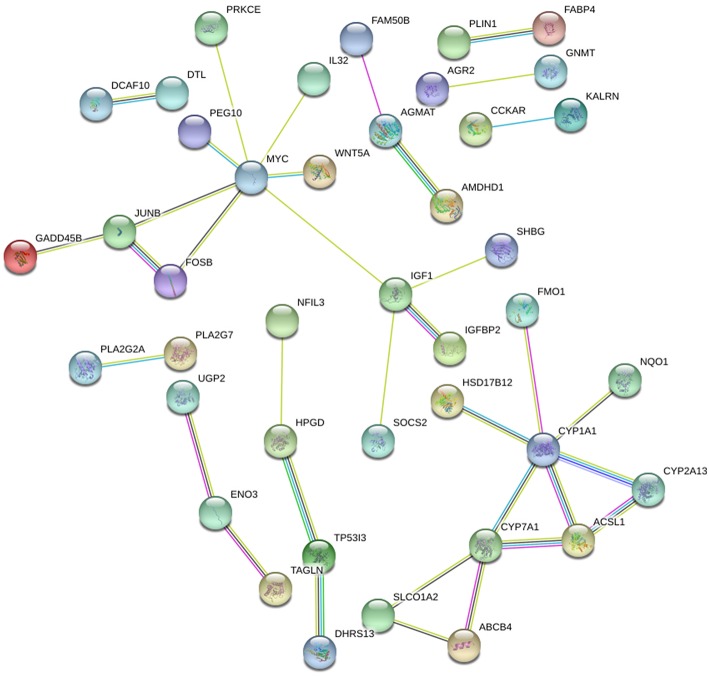
The PPI network of DEGs in NAFLD (STRING). The top 10 genes with most connections included CYP1A1, MYC, CYP7A1, IGF1, JUNB, HPGD, FOSB, ACSL1, ENO3, and CYP2A13.

STRING and HIPPIE database online database was used to perform PPI network analysis of the DEGs and Cytoscape software was adopted to visualize the results. In the PPI analysis, the connections between nodes were visualized to identify the interactions between the proteins encoded by DEGs in NAFLD (Figure 4). The gene located in the central node was considered as the hub gene which may play pivotal physiological regulatory role. In the analysis of STRING, the top 10 genes with most connections were CYP1A1, MYC, CYP7A1, IGF1, JUNB, HPGD, FOSB, ACSL1, ENO3, and CYP2A13. In the analysis of HIPPIE, the top 10 genes were MYC, ENO3, IL32, JUNB, PRKCE, P4HA1, NFIL3, TAGLN, KALRN, and AGMAT. Based on the results of the PPI and the results of the RRA, we finally defined the four most critical genes as the hub genes, including ENO3, CYP7A1, P4HA1, and CYP1A1. The visualization for hub genes in STRING database were shown in Supplementary Figure 3.

### The Validation of RRA and Hub Genes

We validated our RRA results and hub genes using sequencing dataset GSE126848, and found that eight out of the top 10 DEGs found by RRA also had significant differences in the verification dataset. Only SHBG and MAMDC4 did not show significant differences in the validation dataset (*P* > 0.05). In the validation of the top 10 genes in PPI, we found that six of STRING method and eight of HIPPIE method were also identified as DEGs, and all hub genes have been identified as DEGS in validated datasets. The verification results were presented in Supplementary Table 2.

## Discussion

Globally, non-alcoholic fatty liver disease (NAFLD) is the most common chronic liver disease, including a range of pathologies, from benign hepatic steatosis to non-alcoholic steatohepatitis, cirrhosis, and eventually it may develop into hepatocellular carcinoma (16). NAFLD is recognized as a complex disease and the interaction between the environment and the susceptible multi-gene host background determines disease phenotype and progression (17). Therefore, identifying the susceptibility gene of NAFLD is very important for us to study the cause of this disease and to find potential treatments. However, there are still no reports on the use of RRA method to detect DEGs in NAFLD. RRA algorithm, a well-designed tool, has four key features: strong robustness to noise, ability to deal with incomplete ranking, giving significant scores to each element in the result ranking, and high computational efficiency (9). Our study was the first to systematically search and incorporate the microarrays on NAFLD in GEO, which was not available in previous researches. The aim of our study was to identify key genes and their pathways involved in the pathogenesis of NAFLD. In the present study, we included six microarray studies, compared gene expression profiles between NAFLD and controls, adopted the RRA analysis to integrate results with more statistical power. Furthermore, functional annotation and PPI network construction were performed to understand the potential biological function of the DEGs.

In all, 96 DEGs were filtered out across multiple datasets with 50 up-regulated and 46 down-regulated genes. The most significant 10 genes were ENO3, CYP7A1, P4HA1, CYP1A1, IGFBP2, SOCS2, FMO1, PEG10, SHBG, and MAMDC4. Among them, ENO3, CYP7A1, P4HA1, and CYP1A1 were identified as hub genes in PPI network analysis. Enolase 3 (ENO3) encodes the β-subunit of enolase, which is found in skeletal muscle cells in the adult where it may play a role in muscle development and regeneration. Several researches have reported that ENO3 was distributed on various tissues, such as liver, lung, skeletal and heart (18). ENO3 mediates cholesterol ester synthesis, may increase lipid delivery to liver and accelerate hepatic cholesterol ester accumulation (19). In the previous study, Elam et al. used the gene expression profiling and found that ENO3 was significantly higher in livers of morbidly obese women compared with women who had experienced massive weight loss (20). However, the definite function and mechanisms of ENO3 in NAFLD remain unclear.

CYP7A1 and CYP1A1 are both annotated to the pathway of steroid hormone biosynthesis and encode the member of the cytochrome P450 superfamily of enzymes. Cytochrome P450 family seven subfamily A member 1 (CYP7A1) mediates cholesterol metabolism and functions as a rate-limiting enzyme to regulate the process of conversion of cholesterol into bile acids (21, 22). Deficiency of CYP7A1 caused by homozygous deletion mutations can inhibit the production of bile acids, leading to the accumulation of cholesterol in liver, reducing LDL receptors and elevating LDL cholesterol (23). Polymorphism in the promoter of CYP7A1 could affect the synthesis of bile acids and delay the process of lipid responses to the drug fenofibrate (24). CYP7A1 deficiency in mice also showed aberrant changes in the cholesterol and bile acid transformation (25), CYP7A1 transgenic mice represented improved metabolic homeostasis in liver (26, 27). However, our multiple arrays meta-analysis demonstrated that CYP7A1 was up-regulated in NAFLD patients compared with NC. Another research revealed that CYP7A1 and its related cholesterol process were adversely regulated between non-alcoholic fatty liver disease and alcoholic liver disease (28), so it's possible that the increasing CYP7A1 levels in liver tissue are the consequence rather than cause of NAFLD.

Cytochrome P450 family 1 subfamily A member 1 (CYP1A1) encodes a monooxygenase and is widely detected for its ability to activate compounds with carcinogenic properties (29, 30). The enzyme encoded by CYP1A1 catalyze many reactions involved in drug metabolism and synthesis of cholesterol, steroids, and other lipids. Uno et al. indicated that CYP1A1 has a protective role against NAFLD development through experiments *in vivo* (31). Zhu et al. studied the intersection of differentially expressed mRNAs and miRNA-predicted target genes, and then demonstrated that CYP1A1 plays important roles in NAFLD (32). Elam et al. found that CYP1A1 also differentially expressed in liver tissues of morbidly obese patients (20). Therefore, CYP1A1 has a great possibility to be an important gene of NAFLD, but its specific function needs more experimental evidence. In addition, several studies have reported that CYP1A1 polymorphisms were significant factors for the susceptibility and pathogenesis of cancer (33, 34). The contribution of CYP1A1 to cancer progression may be associated with the balance of pre-amino acid activation/detoxification and extrahepatic metabolism of dietary natural products (29).

Our study found that Prolyl-4-hydroxylase α1 (P4HA1) was a hub gene of NAFLD. P4HA1 is a P4H (also called PHD) isoenzyme and an important rate-limiting enzyme. P4H is the primary cellular oxygen sensor and regulates hypoxia-inducible factor (HIF) proteasomal degradation in an oxygen-dependent manner (16). The involvement of P4H in the pathogenesis of NAFLD has been fully proved (35, 36). In NAFLD, adipose tissue expansion and liver fat accumulation impair local oxygen homeostasis. Hypoxia signaling is also a key mechanism of adipose tissue dysfunction, leading to adipose tissue fibrosis, inflammation, and insulin resistance (37). In this case, the body is prone to tissue hypoxia-induced adaptive response. HIF acts as a major regulator of this hypoxic adaptive response, and is further activated by P4H hydroxylation (36–38). Moreover, Seda et al. also found that P4HA1 was significantly differentially expressed in the liver tissue of patients with NAFLD and liver tissue of the control group (39). In addition, it was also significantly differentially expressed in the liver tissue of morbidly obese patients (20).

In our KEGG enrichment analysis, PPAR signaling pathway was significantly enriched and its aberrant expression genes contained CYP7A1, ACSL1, PLIN1, and FABP4 in the DEGs. Per-oxisome proliferator-activated receptors (PPARs) are ligand-activated transcription factors. The PPAR pathway is widely used and involved in a variety of physiological regulation processes, including regulation of cell differentiation and development, involvement in lipid, protein, and carbohydrate metabolism, and tumorigenesis (40, 41). In recent years, PPARs have been identified to play a significant role in modulation of NAFLD due to its involvement in nutrient metabolism (42, 43). There are three PPAR isoforms: α(α), β/δ(β/δ), and γ(γ), which are differentially expressed in tissues (44). PPARα is mainly found in the liver, although expression is also present in other tissues, while PPARγ is highly expressed in adipose tissue (45, 46). It is worth noting that the expression level of PPARγ in liver tissue was significantly increased in patients with NAFLD and experimental models (47–49). Increased PPARγ activity in mouse liver may result in activation of adipogenic gene expression and increased lipid storage in the liver (43). Our study demonstrated that differential genes in liver tissue of NAFLD and control patients were enriched in the PPAR pathway, further demonstrating the importance of the PPAR pathway in the pathology of NAFLD. Additionally, p53 signaling pathway was reported to associated with insulin resistance, even though it showed borderline significance in KEGG analysis (50).

In summary, by using the RRA method we have successfully provided deeper insight to the comprehensive molecular changes in NAFLD pathogenesis, and identified several potential candidate therapeutic targets, including ENO3, CYP7A1, P4HA1, CYP1A1, IGFBP2, SOCS2, FMO1, PEG10, SHBG, and MAMDC4. Among them, ENO3, CYP7A1, P4HA1, and CYP1A1 were defined as hub genes. In addition, through GO and KEGG pathway analysis, we found that these differential genes were mainly enriched in carboxylic acid metabolic process, monocarboxylic acid metabolic process and oxidation-reduction process, and may be involved in steroid hormone biosynthesis and PPAR signaling pathway. However, the underlying molecular mechanisms have not yet been fully elucidated. In the future, more experiments are needed to verify the changes of gene expression and it is necessary to collect a large number of liver tissues from patients with NAFLD and liver tissues from normal controls for additional functional studies.

## Data Availability

The datasets analyzed for this study can be found in the GEO datasets (https://www.ncbi.nlm.nih.gov/gds).

## Author Contributions

All authors listed have made substantial, direct, and intellectual contribution to the work and approved it for publication. Each author acknowledges that he or she participated sufficiently in the work to take public responsibility for its content. All authors of this paper have read and approved the final version.

### Conflict of Interest Statement

The authors declare that the research was conducted in the absence of any commercial or financial relationships that could be construed as a potential conflict of interest.

## Supplementary Material

Supplementary Figure 1 Standardization of the six microarray datasets. The left panel represents the data before standardization, and the right panel represents the standardized data. **(A)** GSE48452, **(B)** GSE63067, **(C)** GSE66676, **(D)** GSE72756, **(E)** GSE89632, **(F)** GSE107231.

Supplementary Figure 2 The KEGG pathway plots. The DEGs were plotted in red color. **(A)** the bubble chart of KEGG result, **(B)** Steroid hormone biosynthesis, **(C)** PPAR signaling pathway. KEGG pathway enrichment analysis showed that steroid hormone biosynthesis (hsa00140; *P*-value = 6.68E-03) and PPAR signaling pathway (hsa03320; *P*-value = 9.95E-03) were significantly enriched.

Supplementary Figure 3 Visualization for hub genes by using the Cytoscape software. Nodes with warm color represent the hub genes in PPI analysis.

Supplementary Table 1 The 96 DEGs identified by RRA method.

Supplementary Table 2 Verification of RRA and hub genes using dataset GSE126848.
