# Supplementary figures and images for: Integrated Analysis of Multiple Microarray Studies to Identify Novel Gene Signatures in Non-alcoholic Fatty Liver Disease
Source: Front Endocrinol (Lausanne). 2019 Aug 30;10:599. doi: 10.3389/fendo.2019.00599 (PMC6736562; doi:10.3389/fendo.2019.00599)

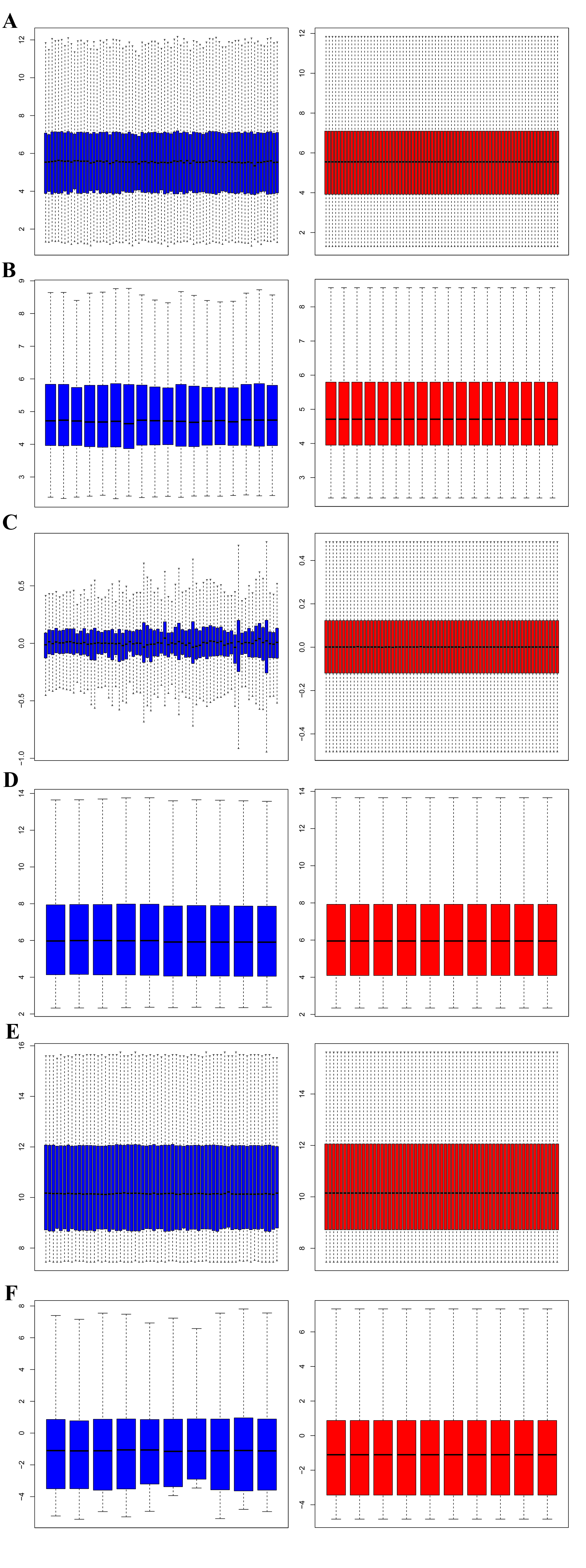

Supplement: Supplementary Figure 1 — Standardization of the six microarray datasets. The left panel represents the data before standardization, and the right panel represents the standardized data. (A) GSE48452, (B) GSE63067, (C) GSE66676, (D) GSE72756, (E) GSE89632, (F) GSE107231. [file Image_1.TIF]

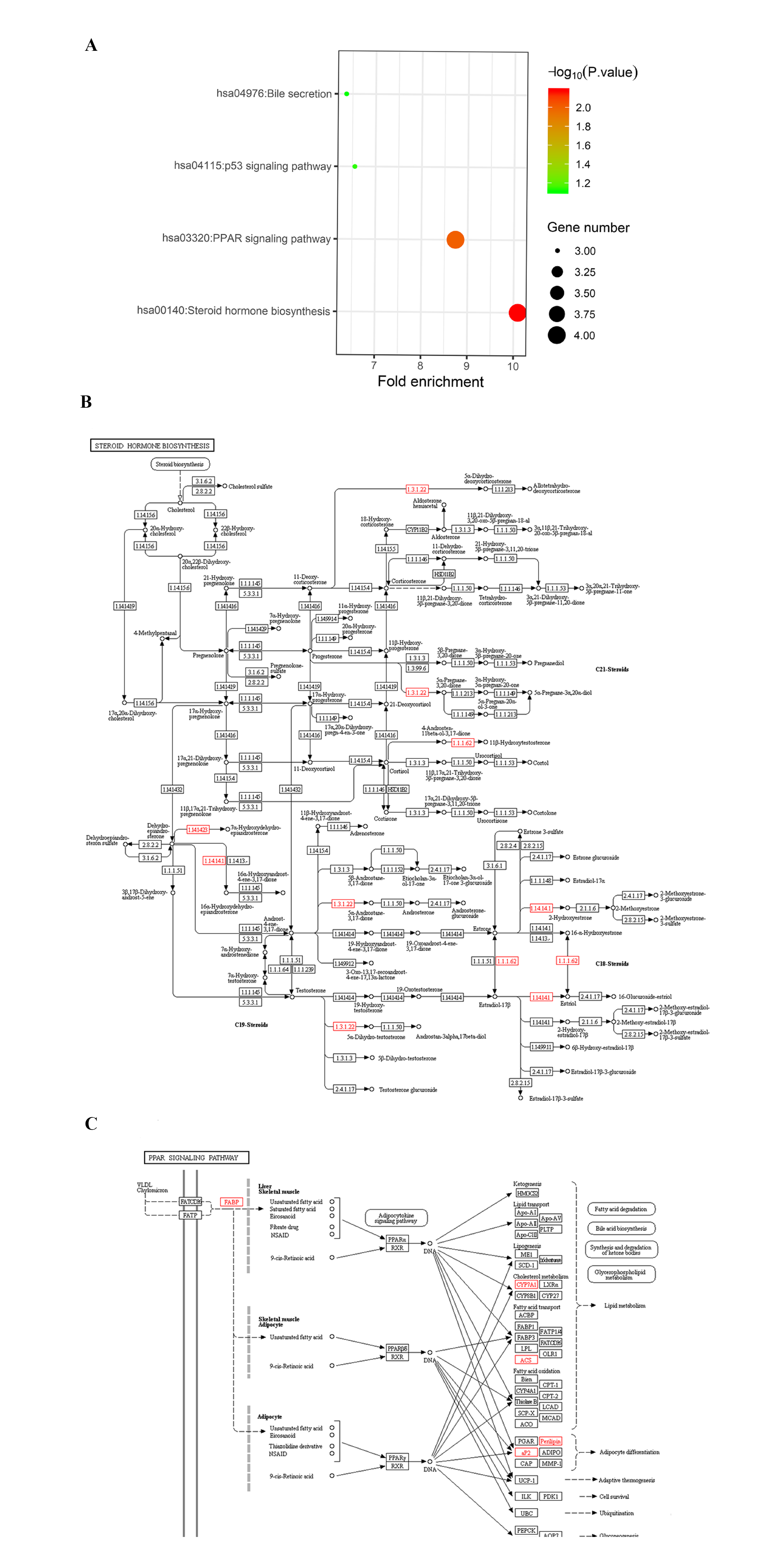

Supplement: Supplementary Figure 2 — The KEGG pathway plots. The DEGs were plotted in red color. (A) the bubble chart of KEGG result, (B) Steroid hormone biosynthesis, (C) PPAR signaling pathway. KEGG pathway enrichment analysis showed that steroid hormone biosynthesis (hsa00140; P-value = 6.68E-03) and PPAR signaling pathway (hsa03320; P-value = 9.95E-03) were significantly enriched. [file Image_2.TIF]

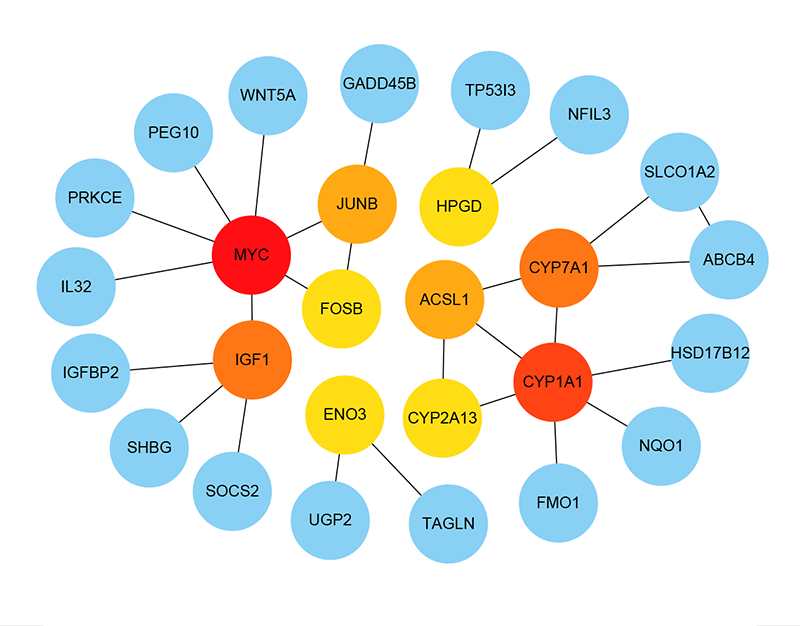

Supplement: Supplementary Figure 3 — Visualization for hub genes by using the Cytoscape software. Nodes with warm color represent the hub genes in PPI analysis. [file Image_3.TIF]
