# Supplementary material for: Integrated Analysis of Multiple Microarray Studies to Identify Novel Gene Signatures in Non-alcoholic Fatty Liver Disease
Source: Front Endocrinol (Lausanne). 2019 Aug 30;10:599. doi: 10.3389/fendo.2019.00599 (PMC6736562; doi:10.3389/fendo.2019.00599)
Supplement: Supplementary Table 1 — The 96 DEGs identified by RRA method. [file Table_1.docx]

| **Supplementary Table 1 The 96 DEGs identified by RRA method.** | | | |
| --- | --- | --- | --- |
| **50 up-regulated DEGs in RRA** | | **46 down-regulated DEGs in RRA** | |
| **Gene symbol** | **P value** | **Gene symbol** | **P value** |
| ENO3 | 7.17E-05 | P4HA1 | 1.67E-04 |
| CYP7A1 | 9.04E-05 | CYP1A1 | 2.51E-04 |
| FMO1 | 6.57E-04 | IGFBP2 | 3.27E-04 |
| PEG10 | 8.95E-04 | SOCS2 | 5.10E-04 |
| MAMDC4 | 1.87E-03 | SHBG | 1.50E-03 |
| TMEM154 | 3.91E-03 | NFIL3 | 7.23E-03 |
| SRD5A2 | 4.45E-03 | C7 | 8.40E-03 |
| IP6K3 | 5.04E-03 | XKR4 | 8.59E-03 |
| CXCL10 | 6.66E-03 | TCN1 | 8.59E-03 |
| IL32 | 7.74E-03 | CFHR4 | 8.59E-03 |
| PLA2G7 | 8.24E-03 | CCDC126 | 8.59E-03 |
| HSD17B12 | 8.24E-03 | FMO4 | 8.59E-03 |
| TM6SF2 | 8.24E-03 | FOSB | 8.59E-03 |
| ITGA8 | 8.24E-03 | SLITRK3 | 1.19E-02 |
| WNT5A | 8.24E-03 | SLCO1A2 | 1.45E-02 |
| DTL | 8.50E-03 | RTN2 | 1.72E-02 |
| CLDN1 | 1.06E-02 | LGALS2 | 1.72E-02 |
| C1QTNF7 | 1.65E-02 | CADM2 | 1.72E-02 |
| ACSL1 | 1.65E-02 | KALRN | 1.72E-02 |
| BBOX1 | 1.65E-02 | FAM50B | 1.72E-02 |
| PRKCE | 1.65E-02 | MYC | 1.72E-02 |
| KCNN4 | 1.65E-02 | ARL14 | 1.72E-02 |
| AKTIP | 1.65E-02 | TSPAN8 | 1.84E-02 |
| SUPT5H | 2.47E-02 | AMDHD1 | 1.84E-02 |
| PLA2G2A | 2.47E-02 | DHRS13 | 2.34E-02 |
| TP53I3 | 2.47E-02 | PPM1K | 2.40E-02 |
| COTL1 | 2.47E-02 | SBSN | 2.47E-02 |
| PLIN1 | 2.47E-02 | CCKAR | 2.57E-02 |
| NAT8B | 2.47E-02 | SLIT3 | 2.57E-02 |
| CAPN12 | 2.54E-02 | VIL1 | 2.57E-02 |
| HPGD | 2.60E-02 | THAP10 | 2.57E-02 |
| PPP1R3C | 2.67E-02 | JUNB | 2.57E-02 |
| SLC41A2 | 2.81E-02 | TSPAN13 | 2.60E-02 |
| KDM1B | 3.29E-02 | IGF1 | 2.97E-02 |
| UGP2 | 3.29E-02 | TMED6 | 3.27E-02 |
| AGMAT | 3.29E-02 | IMPDH1 | 3.43E-02 |
| TFF2 | 3.29E-02 | NQO1 | 3.43E-02 |
| TMEM169 | 3.29E-02 | STOX1 | 3.43E-02 |
| PALMD | 3.54E-02 | GADD45B | 3.43E-02 |
| RPL35A | 4.11E-02 | SRPX | 3.76E-02 |
| KIAA1324L | 4.11E-02 | GNMT | 3.81E-02 |
| ABCB4 | 4.11E-02 | TAGLN | 3.88E-02 |
| TRPM8 | 4.11E-02 | AGR2 | 4.29E-02 |
| UNC93A | 4.79E-02 | DCAF10 | 4.29E-02 |
| ERP29 | 4.93E-02 | DYNLL1 | 4.29E-02 |
| FABP4 | 4.93E-02 | FAM107A | 4.29E-02 |
| CYP2A13 | 4.93E-02 |  |  |
| MRAS | 4.93E-02 |  |  |
| SSFA2 | 4.93E-02 |  |  |
| RNF43 | 4.93E-02 |  |  |
