# Supplementary material for: Integrated Analysis of Multiple Microarray Studies to Identify Novel Gene Signatures in Non-alcoholic Fatty Liver Disease
Source: Front Endocrinol (Lausanne). 2019 Aug 30;10:599. doi: 10.3389/fendo.2019.00599 (PMC6736562; doi:10.3389/fendo.2019.00599)
Supplement: Supplementary Table 2 — Verification of RRA and hub genes using dataset GSE126848. [file Table_2.docx]

**Supplementary Table 2** **Verification of RRA and hub genes using dataset GSE126848**

| **Validation of RRA** | | **Validation of the hub gene of STRING** | | **Validation of the hub gene of HIPPIE** | |
| --- | --- | --- | --- | --- | --- |
| **Gene symbol** | **P value** | **Gene symbol** | **P value** | **Gene symbol** | **P value** |
| ENO3 | 9.85E-07 | CYP1A1 | 2.77E-03 | MYC | 4.48E-02 |
| CYP7A1 | 2.22E-04 | MYC | 4.47E-02 | ENO3 | 9.85E-07 |
| P4HA1 | 5.37E-33 | CYP7A1 | 2.22E-04 | IL32 | 2.58E-11 |
| CYP1A1 | 2.77E-03 | IGF1R | 7.24E-08 | JUNB | 3.67E-01 |
| IGFBP2 | 7.13E-15 | JUNB | 3.67E-01 | PRKCE | 6.01E-14 |
| SOCS2 | 3.30E-10 | HPGD | 9.74E-04 | P4HA1 | 5.37E-33 |
| FMO1 | 3.01E-09 | FOSB | 9.25E-01 | NFIL3 | 2.43E-01 |
| PEG10 | 7.97E-09 | ACSL1 | 3.24E-01 | TAGLN | 1.32E-03 |
| SHBG | 8.35E-01 | ENO3 | 9.85E-07 | KALRN | 2.71E-09 |
| MAMDC4 | 9.77E-01 | CYP2A13 | 4.76E-01 | AGMAT | 1.92E-09 |
